# Initial results from a multi-center population-based cluster randomized trial of esophageal and gastric cancer screening in China

**DOI:** 10.1186/s12876-020-01517-3

**Published:** 2020-11-24

**Authors:** Hongmei Zeng, Kexin Sun, Maomao Cao, Rongshou Zheng, Xibin Sun, Shuzheng Liu, Zhiyi Zhang, Yuqin Liu, Guizhou Guo, Guohui Song, Yigong Zhu, Xianghong Wu, Bingbing Song, Xianzhen Liao, Yanfang Chen, Mingyang Song, Edward Giovannucci, Guihua Zhuang, Wenqiang Wei, Wanqing Chen, Jie He

**Affiliations:** 1 National Central Cancer Registry, National Cancer Center/National Clinical Research Center for Cancer/Cancer Hospital, Chinese Academy of Medical Sciences and Peking Union Medical College, Beijing, China; 2 Office for Cancer Screening, National Cancer Center/National Clinical Research Center for Cancer/Cancer Hospital, Chinese Academy of Medical Sciences and Peking Union Medical College, 17 South Lane, Panjiayuan, Chaoyang District, Beijing, 100021 China; 3 Henan Office for Cancer Control and Research, The Affiliated Cancer Hospital of Zhengzhou University, Henan Cancer Hospital, Zhengzhou, China; 4 Department of Gastroenterology, Gansu Wuwei Tumor Hospital, Wuwei, China; 5 Cancer Epidemiology Research Center, Gansu Provincial Cancer Hospital, Lanzhou, China; 6 Linzhou Cancer Hospital, Linzhou, China; 7 Cixian Cancer Institute, Handan, China; 8 Luoshan Center for Disease Control and Prevention, Xinyang, China; 9 Center for Disease Control and Prevention of Sheyang County, Yancheng, China; 10 Institute of Cancer Prevention and Treatment, Harbin Medical University, Harbin, China; 11 Hunan Office for Cancer Control and Research, Hunan Cancer Hospital, Changsha, China; 12 Yueyang Lou District Center for Disease Prevention and Control, Yueyang, China; 13 Department of Epidemiology, Harvard T.H. Chan School of Public Health, Boston, USA; 14 Department of Nutrition, Harvard T.H. Chan School of Public Health, Boston, USA; 15 Clinical and Translational Epidemiology Unit, Massachusetts General Hospital and Harvard Medical School, Boston, USA; 16 Division of Gastroenterology, Massachusetts General Hospital and Harvard Medical School, Boston, USA; 17 Department of Epidemiology and Biostatistics, School of Public Health, Xi’an Jiaotong University Health Science Center, Xi’an, China; 18 Department of Thoracic Surgery, National Cancer Center/National Clinical Research Center for Cancer/Cancer Hospital, Chinese Academy of Medical Sciences and Peking Union Medical College, Beijing, China

**Keywords:** Esophageal cancer, Gastric cancer, Endoscopic screening, Trial, China

## Abstract

**Background:**

We initiated the first multi-center cluster randomized trial of endoscopic screening for esophageal cancer and gastric cancer in China. The objective of the study was to report the baseline screening findings in this trial.

**Methods:**

We recruited a total of 345 eligible clusters from seven screening centers. In the intervention group, participants from high-risk areas were screened by endoscopy; in non-high-risk areas, high-risk individuals were identified using a questionnaire and advised for endoscopy. Lugol’s iodine staining in esophagus and indigo carmine dye in stomach were performed to aid in the diagnosis of suspicious lesions. The primary outcomes of this study were the detection rate (proportion of positive cases among individuals who underwent endoscopic screening) and early detection rate (the proportion of positive cases with stage 0/I among all positive cases).

**Results:**

A total of 149,956 eligible subjects were included. The detection rate was 0.7% in esophagus and 0.8% in stomach, respectively. Compared with non-high-risk areas, the detection rates in high-risk areas were higher, both in esophagus (0.9% vs. 0.1%) and in stomach (0.9% vs. 0.3%). The same difference was found for early-detection rate (esophagus: 92.9% vs. 53.3%; stomach: 81.5% vs. 33.3%).

**Conclusions:**

The diagnostic yield of both esophagus and stomach were higher in high-risk areas than in non-high-risk areas, even though in non-high-risk areas, only high-risk individuals were screened. Our study may provide important clues for evaluating and improving the effectiveness of upper-endoscopic screening in China.

*Trial registration*: Protocol Registration System in Chinese Clinical Trial Registry, ChiCTR-EOR-16008577. Registered 01 June 2016-Retrospectively registered, http://www.chictr.org.cn/showprojen.aspx?proj=14372

## Background

China is a high-risk country for esophageal cancer (EC) and gastric cancer (GC), accounting for about 50% (763,483 new cases, 673,615 deaths in 2018) of the global burden [1]. Most EC and GC cases present in advanced stages and the overall 5-year survival is low. Early detection and treatment have great potential to improve survival and reduce disease mortality.

Upper endoscopy with biopsy is the gold-standard for diagnosis of EC and GC. In Japan and South Korea, endoscopy has been widely used as a screening method for GC [2, 3]. The upper endoscopy allows for examination of the entire esophagus and stomach with one-time endoscopy. In the real-world cancer screening practices currently conducted in China, we screen both EC and GC using upper endoscopy. However, distinct practical strategies are adopted in different areas of China. For the *Cancer Screening Program in Rural Areas*, a massive screening method on EC and GC has been conducted in high-risk areas of China [4]. For the *Cancer Screening Program in Huai River Areas* and *Cancer Screening Program in Urban Areas*, subjects are first evaluated with a questionnaire and those who are classified as high-risk individuals will be further screened by endoscopy.

Many studies showed that endoscopic screening might be associated with reduced mortality on EC and GC in some high-risk areas of Asia [4–8]. Considering the fact that striking geographic variations exist in the incidences of EC and GC within China, which can vary more than tenfold in different regions [9], whether endoscopic screening remains a cost-effective method with reduced mortality on EC and GC in areas with non-high risk were not well known. And high-level evidence evaluating the efficacy of screening on EC and GC in different areas of China is still lacking. Therefore, to evaluate the efficacy and feasibility of endoscopic screening on prevention of EC and GC in different areas of China, we initiated a population-based, cluster randomized controlled trial in May 2015, covering three high-risk areas and four non-high-risk areas of the country. Such evidence is valuable in guiding esophageal and stomach cancer control policies. The main aim of the trial was to evaluate the efficacy of upper endoscopic screening in reducing mortality of EC and GC in different regions of China; we also aimed to establish a large database and biobank within the trial for further research. By July 2017, baseline investigation of the trial has been completed. A multi-center large cancer screening cohort, as well as a database and a biobank have been established. In the present study, we reported the demographics and other baseline characteristics of this population, compliance of endoscopic screening, yield of the first-round endoscopic screening and major complications in this community-based randomized trial covering a total of 149,956 individuals in China.

## Methods

### Trial design

The study design has been described in detail previously [10]. We selected three high-risk areas (Cixian, Linzhou, Wuwei) and four non-high-risk areas (Changsha, Harbin, Luoshan, Sheyang) as screening centers in our study. According to the definition established in the First National Death Survey in 1973–1975 [9], if the crude mortality rate of EC during 1973–1975 was more than 60/100,000 in males and 30/100,000 in females, the areas (such as Linzhou and Cixian) would be classified as high-risk areas of esophageal cancer. If the crude mortality rate of GC was more than 50/100,000 in males and 25/100,000 in females, the areas would be classified as areas with high risk of GC (such as Wuwei).

A total of 345 eligible villages/communities in seven screening centers which met the criteria of cluster inclusion constituted the randomization unit. Clusters which had implemented endoscopic screening in the latest 3 years and those who are unwilling to participate were excluded. We used stratified cluster sampling design to assign those clusters to two groups in 1:1 ratio by each center, due to practical reasons and contamination prevention. We randomly assigned the clusters into the intervention group or control group. The local village doctors or community public health workers in each site recruited participants to each group according to the study-group assignment. Both local doctors and study subjects were aware of the study-group assignment. The study was initiated in May 2015 and the recruitment was finished in July 2017. No major changes to methods after trial commencement had been made.

### Participants

Eligible participants were residents aged 40–69 years old, had no personal history of cancer, had not received endoscopy in the past 3 years. After explaining the study and obtaining written informed consent, local health workers had a face-to-face interview with the subjects using a computer-aided standardized questionnaire. The participants also received normal community care including measurement of blood pressure, weight, and height.

### Intervention

In the intervention group, participants from high-risk areas were automatically identified as high-risk individuals and were invited to receive upper endoscopy; in non-high-risk areas, they were evaluated with a risk assessment questionnaire, which has been widely used in the current screening practice in non-high-risk areas of China. The questionnaire used to define high-risk individuals is shown in Additional file 1: Table S1. Only subjects who were identified as high-risk individuals were invited to receive endoscopy in the intervention group of non-high-risk areas. Before endoscopy, a 4-mL blood sample was collected to screen for hepatitis B virus, hepatitis C virus, Human Immunodeficiency Virus and syphilis; participants with any of these infections were allocated to use a special endoscope with very strict cleaning and disinfection requirement. Another 10-mL blood sample for each subject was collected and separated for plasma and white blood cells before endoscopic screening. The plasma and white blood cells were placed in − 80 °C freezer for long-term preservation for biobank construction.

High-risk individuals in the screening arm received standard upper endoscopy by experienced physicians. The entire esophagus and stomach were visually examined. Lugols’ iodine staining in esophagus and indigo carmine dye in stomach were performed when necessary to aid in the diagnosis of suspicious lesions. Suspicious lesions were targeted for biopsy for further pathological diagnosis. Subjects without suspicious lesions did not receive a biopsy.

### Histological categories

The biopsy slides were independently reviewed by two experienced pathologists and discrepancies in their diagnoses were adjudicated by consultation. About 1% slides were randomly selected and blindly reviewed by an external senior pathologist from NCC China and the consensus was more than 98%. Histological criteria using WHO classification have been described previously [11]. The following diagnosis was classified as positive cases: (1) esophageal squamous severe dysplasia, (2) EC in situ, (3) EC, (4) gastric high-grade dysplasia, (5) GC. Subjects with squamous severe dysplasia, EC in situ, gastric high-grade dysplasia, or EC/GC in stage I were identified as positive cases at an early stage.

### Follow-up and outcome ascertainment

After finishing the intervention, we continue to follow up the study population using passive follow-up and active follow-up annually. For passive follow-up, we link the identifiable information with the data from local cancer registries, vital system, medical insurance database, and clinical settings to identify new cancer cases and deaths in the cohort. Cancer cases identified through passive follow-up would further be interviewed by telephone, home visit or local public health workers for their conditions and detailed diagnosis and treatment regimens. The follow-up is ongoing and will continue at least up to 10 years to evaluate the long-term effects of endoscopic screening.

### Outcome measure

The primary outcome of the trial was EC/GC mortality. The outcome measures of the current study were overall response proportion, detection rate, stage distribution, treatment rate, and major complication rate. The response proportion was calculated as the proportion of subjects who finished questionnaire among subjects invited in the study. The detection rate was calculated as the proportion of positive cases among subjects who underwent endoscopy. The early-detection rate was calculated as the proportion of positive cases with stage 0/I among all positive cases. The treatment rate was calculated as the proportion of positive cases who received any clinical treatment (i.e. endoscopic therapy, surgery, chemotherapy, radiotherapy or palliative care). The definition of major complication rate was the number of major complications (i.e. bleeding, perforation, gastrospasm requiring medical management) relative to the number of individuals who underwent endoscopy.

### Statistical analysis

Research data were reported to National Cancer Center of China. Data quality control was performed according to standard data management procedures by two experienced statisticians. For description of baseline characteristics, continuous variables were described using mean and standard deviation (SD). Categorical variables were described as frequency and proportion. Chi-square test or Fisher’s exact test was used to compare categorical variables across groups when applicable. To explore the factors which may be associated with detection rates and early-detection rates, we used multivariate unconditional Logistic regression, with adjustment for age, sex and participating center. With an alpha 0.05, the trial has 80% power to detect a 30% absolute decrease in mortality rate of EC and GC in high-risk areas and a 25% absolute decrease in mortality rate in non-high-risk areas after 10 years’ follow-up. Two-sided *P* < 0.05 were considered statistically significant. Statistical analyses were performed in SAS version 9.4 (SAS Institute, Cary, NC).

## Results

### Study population and participation

**Fig. 1:**
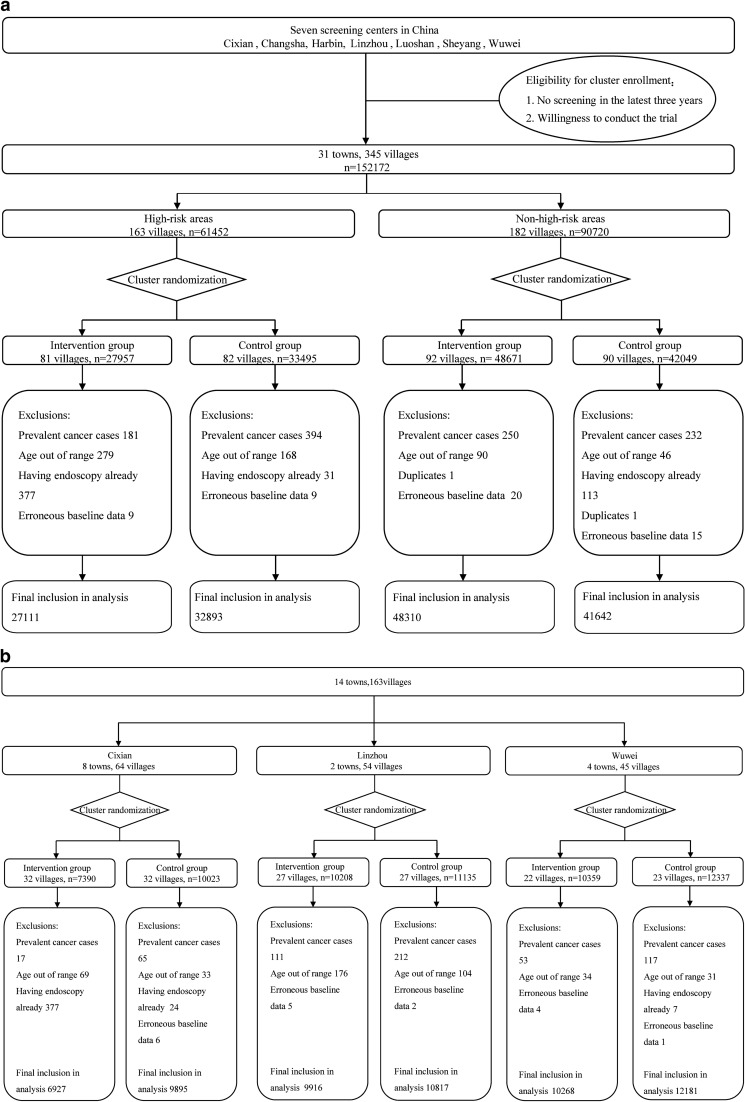

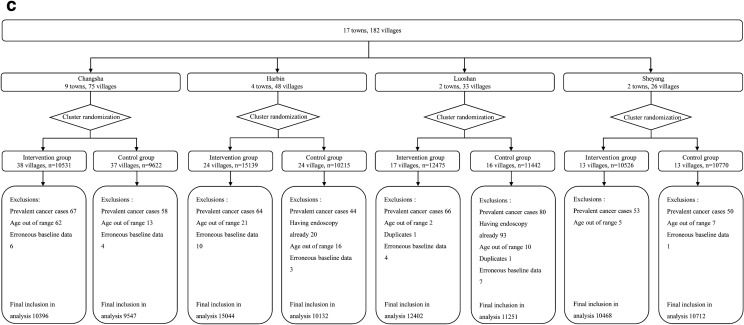
Flow diagram of the participants in the multi-center randomized trial, overall and by areas: **a** Overall. **b** High-risk areas. **c** Non-high-risk areas

The incidence rates of EC and GC varied greatly among seven screening centers in our study (Additional file 1: Table S2). Overall, 230,583 subjects from a total of 345 eligible clusters in these screening centers were invited to the trial and 152,172 people attended the baseline survey between May 2015 and July 2017, with an overall response proportion of 66.0%. The response proportion in the intervention group (62.0%) was significantly lower than that in the control group (70.6%) (*P* < 0.001). Of 152,172 participants, we excluded 2216 (1.5%) participants with personal history of cancer (n = 1057), age out of range (n = 583), previous history of endoscopic screening (n = 521) or duplicates/erroneous baseline data (n = 55). The final cohort eligible for analysis consisted of 149,956 individuals (75,421 in the intervention group and 74,535 in the control group) (Fig. 1a). In high-risk areas, there were 27,111 people in the intervention group and 32,893 in the control group (Fig. 1b); in non-high-risk areas, there were 48,310 subjects in the intervention group and 41,642 in the control group (Fig. 1c). Baseline characteristics were balanced between study groups (Table 1), regarding both mean age (SD) 53.8 (7.9) in the intervention group and 53.46 (8.1) in the control group, and the proportion of women subjects (55.8% in the intervention group and 54.1% in the control group). The distribution of education background, smoking and drinking status was also similar between the intervention group and control group, overall and by area.

**Table 1:** Baseline characteristics of participants in the different groups

Variables	All (%)		High-risk areas (%)		Non-high-risk areas (%)	
	Intervention group	Control group	Intervention group	Control group	Intervention group	Control group
Sex						
Male	44.2	45.9	43.0	46.2	45.0	45.7
Female	55.8	54.1	57.0	53.8	55.0	54.3
Age group						
60–69	28.7	28.1	25.4	27.8	30.7	28.2
50–59	36.9	35.0	37.7	34.3	36.4	35.6
40–49	34.4	36.9	36.9	37.9	32.9	36.2
Ethnicity						
Han	99.6	99.8	99.9	99.9	99.5	99.6
Others	0.4	0.2	0.1	0.1	0.5	0.4
Marriage						
Married	94.5	95.3	94.1	95.4	94.7	95.2
Never married	0.9	0.8	0.6	0.5	1.1	1.1
Divorced	0.9	0.7	0.4	0.4	1.1	0.9
Widow	3.7	3.2	4.9	3.7	3.1	2.8
Education						
No schooling	10.3	9.5	16.8	14.1	6.6	6.0
Primary school	33.6	37.8	34.3	42.2	33.1	34.2
Middle school	46.4	45.3	47.7	42.9	45.7	47.3
College and above	9.7	7.3	1.2	0.8	14.6	12.5
Smoking						
Never	79.0	78.1	74.1	74.4	81.7	80.9
Ever	21.0	21.9	25.9	25.6	18.3	19.1
Alcohol drinking						
No	87.3	88.4	88.2	90.7	86.7	86.6
Yes	12.7	11.6	11.8	9.3	13.3	13.4

### Screening findings

In high-risk areas, all eligible subjects in the intervention group were invited to undergo endoscopy unless the subjects were not able to attend due to health conditions. The overall compliance rate for endoscopy was 43.8%. In high-risk areas, 27,111 eligible participants finished questionnaire and 26,633 underwent endoscopy. The compliance rate for endoscopy in high-risk areas was 42.2% (denominator 63,123 eligible individuals invited). In non-high-risk areas, 48,310 eligible subjects finished questionnaire in the intervention group and 23,532 were identified as high-risk individuals for further endoscopy; and 11,289 (compliance rate 48.0%, denominator 23,532 high-risk individuals invited) subjects finished endoscopy.

Among 37,922 subjects who underwent endoscopy, 528 (detection rate: 1.4%) positive cases were detected. The detection rate was higher in high-risk areas (1.8%) than in non-high-risk areas (0.4%). The detailed pathological distribution of all screening subjects is shown in Table 2. In esophageal site, the prevalence of esophagitis, mild/moderate dysplasia/dysplasia NOS, severe dysplasia/EC in situ, and EC were 11.5%, 4.5%, 0.5% and 0.2% respectively. In stomach site, the prevalence for atrophic gastritis, intestinal metaplasia/low-grade dysplasia, high-grade dysplasia and GC was 14.6%, 7.9%, 0.3%, and 0.4% respectively. We consistently found the statistically higher prevalence of advanced lesions and precancerous lesions in high-risk areas than that in non-high-risk areas (Table 2).

**Table 2:** Prevalence of different lesions according to the per-protocol analysis

Pathological diagnosis	Cases *N* (%)	High-risk areas *N* (%)	Non-high-risk areas *N* (%)	*P* value
All subjects under endoscopy	37,922	26,633	11,289	
Esophagus				
Esophagitis	4349 (11.5)	4173 (15.7)	176 (1.6)	< 0.001
Mild/moderate dysplasia, or dysplasia, NOS	1692 (4.5)	1600 (6.0)	92 (0.8)	< 0.001
Severe dysplasia/EC in situ	195 (0.5)	187 (0.7)	8 (0.1)	< 0.001
EC	59 (0.2)	52 (0.2)	7 (0.1)	0.003
Positive esophageal lesions	254 (0.7)	239 (0.9)	15 (0.1)	< 0.001
Stomach				
Atrophic gastritis	5522 (14.6)	5132 (19.3)	390 (3.5)	< 0.001
Intestinal metaplasia/low-grade dysplasia	2977 (7.9)	2257 (8.5)	720 (6.4)	< 0.001
High-grade dysplasia	117 (0.3)	105 (0.4)	12 (0.1)	< 0.001
GC	167 (0.4)	143 (0.5)	24 (0.2)	< 0.001
Positive stomach lesions	284 (0.8)	248 (0.9)	36 (0.3)	< 0.001
Positive cases in total^a^	528 (1.4)	478 (1.8)	50 (0.4)	< 0.001

^**a**^Ten positive cases had advanced lesions both at esophageal and stomach site

**Fig. 2:**
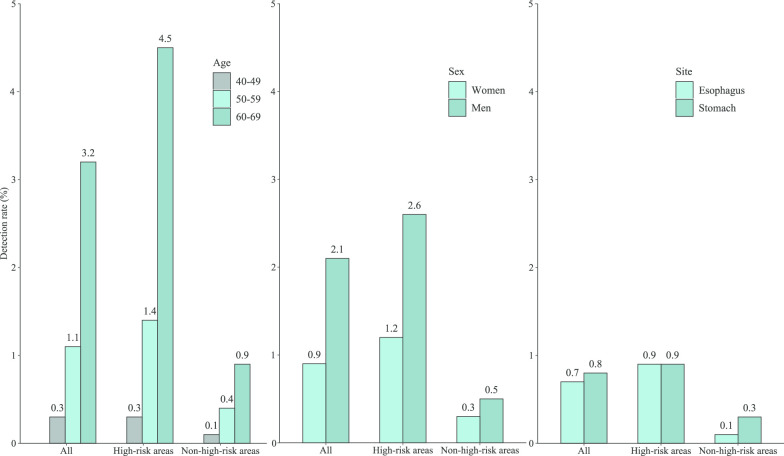
The detection rates of participants in high-risk areas and non-high-risk areas

We explored possible factors which may affect the detection rates in endoscopic screening (Table 3, Fig. 2). We found that the detection rates in subjects aged 50–59 and 60–69 years were 3.2- and 12.3-times higher than those in their 40 s (*P* trend < 0.001). Men had a higher detection rate than women (odds ratio [OR]:2.2, 95% confidence interval [CI]:1.9–2.7). The detection rate in high-risk areas was more than three times higher than in non-high-risk areas (OR: 4.9, 95% CI: 3.7–6.6). We further explored the detection rates by anatomical site and consistently found a higher detection rate in the elderly than those who were young, in men than those in women, in high-risk areas than those in non-high-risk areas.

**Table 3:** Factors associated with detection rates, overall and by site

Site	Total	Positive cases (*N*)	Detection rate (%)	OR (95% CI) ^a^
Esophagus and stomach	Age, years			
	40–49	36	0.3	1
	50–59	156	1.1	4.2 (2.9–6.0)
	60–69	336	3.2	13.3 (9.4–18.8)
	Sex			
	Women	195	0.9	1
	Men	333	2.1	2.2 (1.9–2.7)
	Area			
	Non-high-risk	50	0.4	1
	High-risk	478	1.8	4.9 (3.7–6.6)
Esophagus	Age, years			
	40–49	10	0.1	1
	50–59	66	0.5	6.4 (3.3–12.4)
	60–69	178	1.7	25.6 (13.5–48.4)
	Sex			
	Women	111	0.5	1
	Men	143	0.9	1.6 (1.3–2.1)
	Area			
	Non-high-risk	15	0.1	1
	High-risk	239	0.9	8.2 (4.9–13.9)
Stomach	Age, years			
	40–49	26	0.2	1
	50–59	91	0.6	3.3 (2.2–5.1)
	60–69	167	1.6	8.7 (5.8–13.2)
	Sex			
	Women	86	0.4	1
	Men	198	1.2	3.0 (2.3–3.9)
	Area			
	Non-high-risk	36	0.3	1
	High-risk	248	0.9	3.4 (2.4–4.8)

^a^Adjusted for age, sex, and screening center

### Clinical characteristics of positive cases

Table 4 shows the detailed stage distribution of positive cases in the screening cohort. The early-detection rate was 82.6% overall. By area, the early-detection rate was higher in high-risk areas than in non-high-risk areas (87.0% vs. 40.0%, *P* < 0.001), in esophagus than in stomach (90.6% vs. 75.4%, *P* < 0.001).

**Table 4:** Stage distribution and treatment rate for positive cases screened from the cohort

Site	Variable	*N* (%)	High-risk areas	Non-high-risk areas	*P* value
			*N* (%)	*N* (%)	
All (n = 528)	Stage				< 0.001
	0	258 (48.9)	247 (51.7)	11 (22.0)	
	I	178 (33.7)	169 (35.3)	9 (18.0)	
	II	46 (8.7)	32 (6.7)	14 (28.0)	
	III/IV	46 (8.7)	30 (6.3)	16 (32.0)	
	Early-stage^a^				< 0.001
	No	92 (17.4)	62 (13.0)	30 (60.0)	
	Yes	436 (82.6)	416 (87.0)	20 (40.0)	
	Treatment				0.511
	No	103 (19.5)	95 (19.9)	8 (16.0)	
	Yes	425 (80.5)	383 (80.1)	42 (84.0)	
Esophagus (n = 254)	Stage				< 0.001
	0	166 (65.4)	160 (67.0)	6(40.1)	
	I	64 (25.2)	62 (25.9)	2(13.3)	
	II	17 (6.6)	12 (5.0)	5(33.3)	
	III/IV	7 (2.8)	5 (2.1)	2(13.3)	
	Early-stage^a^				< 0.001
	No	24 (9.4)	17 (7.1)	7 (46.7)	
	Yes	230 (90.6)	222 (92.9)	8 (53.3)	
	Treatment				0.271
	No	44 (17.3)	42 (17.6)	2(13.3)	
	Yes	210 (82.7)	197 (82.4)	13(86.7)	
Stomach (n = 284)	Stage				< 0.001
	0	96 (33.8)	91 (36.7)	5 (13.9)	
	I	118 (41.6)	111 (44.7)	7 (19.4)	
	II	31 (10.9)	21 (8.5)	10 (27.8)	
	III/IV	39 (13.7)	25 (10.1)	14 (38.9)	
	Early-stage^a^				< 0.001
	No	70 (24.6)	46 (18.5)	24 (66.7)	
	Yes	214 (75.4)	202(81.5)	12 (33.3)	
	Treatment				0.452
	No	61 (21.5)	55 (22.2)	6 (16.7)	
	Yes	223 (78.5)	193 (77.8)	30 (83.3)	

^**a**^EC/GC cases with stage 0/I

The overall treatment rate was 80.5% among positive cases. The proportions for subjects receiving surgery, radiotherapy and chemotherapy as initial treatment were 72.3%, 1.5% and 2.1% respectively (Additional file 1: Table S3). The treatment rates for esophageal positive cases and gastric positive cases were 82.7% and 78.5%, respectively (*P* = 0.225). No statistical significance was found for the treatment rates between high-risk areas and non-high-risk areas (80.1% vs. 84.0%). However, the early stage proportion for subjects who did not receive treatment was significantly higher than those who received treatment (early-stage proportion: 95.1% vs. 79.5%, *P* < 0.001).

### Complications

A total of 12 cases (0.3 per 1000) had complications of endoscopy (8 cases with bleeding, 2 with esophageal perforation, 1 with gastric perforation, 1 with gastrospasm). The complication rate was higher in non-high-risk areas (0.5 per 1000) than in high-risk areas (0.2 per 1000) (*P* < 0.001). All subjects with complications were treated timely and no death occurred due to complications.

## Discussion

To the best of our knowledge, screening for EC and GC has not been assessed in a randomized controlled trial in China. The present study reported the feasibility and initial results of a cluster randomized trial of endoscopic screening for EC and GC in China. We found that the detection rate was higher in high-risk areas than in non-high-risk areas, in males than in females, in the elderly than those who were young. Meanwhile, the early-detection rate was higher in esophagus than in stomach, in high-risk areas than in non-high-risk areas of China. Our study may provide important clues for evaluating effectiveness of EC and GC screening in China. Meanwhile, the results suggest that we need to further improve the diagnostic yield especially in non-high-risk areas in the future.

In our study, high-risk areas were determined by different incidence rates of EC and GC. The high-risk areas covered in our study have among the highest incidence of EC and GC in the world [1]. For instance, in Cixian, the age-standardized rate (ASR) of incidence by world Segi’s population for EC was 113.6 per 100,000 in males and 63.4 per 100,000 in females; whereas in Wuwei, the ASR for GC was as high as 138.7 per 100,000 in males and 39.7 per 100,000 in females. Based on the findings that high-risk areas had higher detection rate than non-high-risk areas, we may conclude that these designations are valid. Since 2005, the Chinese government has implemented a series of screening and early detection programs, initially in 11 high-risk areas in 2005, which later expanded to all provinces of mainland China in 2019 [12]. Public education campaign on the benefit of endoscopic screening has been ongoing for more than 10 years in the high-risk areas, which may explain higher compliance of endoscopy as part of a general physical examination in those areas in our study than the previous reports (19.1–27.1%) [13, 14]. Our results showed a higher detection rate (1.8%) in high-risk areas of China than previous upper endoscopic screening studies (0.3–1.3%) [15–17], and a higher early-detection rate in esophagus (90.6%) and in stomach (75.4%) than previous findings [3, 16]. We also observed a lower complication rate in high-risk areas than in non-high-risk areas and in previous reports [18]. The study design of both cancers’ screening, the high-risk exposure of the population living in the areas which results in high prevalence of the diseases, and the extensive training of endoscopic techniques were potential explanatory factors for the high yield of screening results in these high-risk areas [19].

Our previous study conducted in high-risk areas of China found that the early-stage distribution for cases identified from screening cohorts was much higher than the cancer patients who were not attending the screening [20]. One-time screening may reduce EC mortality and be cost-effective in these areas [5, 6]. A prospective study of endoscopic screening in high-risk populations with a small sample size reported no reductions in GC mortality in China [21]. However, many other studies showed endoscopic screening may be associated with reduced GC mortality in Japan, South Korea and rural China [18, 22, 23]. The preliminary findings of our trial add further evidence on the feasibility and efficacy of endoscopic screening in high-risk areas of China. Additional cases of EC/GC might be detected during the ongoing re-screening program for cancer precursors. Meanwhile, more tumors might have been prevented in the endoscopy group owing to the larger number of precancerous diseases detected and treated. There would be a potential advantage of this strategy in terms of reducing not only the rate of death from EC/GC but also the incidence of the diseases in high-risk areas of China.

Endoscopy may not be a practical strategy for mass screening in non-high-risk areas of China because of its expense and invasive nature as well as the low prevalence of the diseases. In non-high-risk areas, only high-risk individuals were screened in our study. We found the detection rates of non-early-stage cancer did not show significance between high-risk areas and non-high-risk areas. However, we found a significantly lower early-detection rate in non-high-risk areas than those in high-risk areas that adopted a mass population endoscopic strategy. The potential explanations include: (1) the risk assessment tool we used in non-high-risk areas might be able to identify late stage, but might not be accurate enough to identify high-risk participants. (2) Individuals’ awareness of endoscopic screening may affect subjects’ participation of screening. Since most EC and GC cases are asymptomatic for a long period, only 20–30% of patients who seek medical advice after developing symptoms are diagnosed at early stages. In those non-high-risk areas, many people fear physical discomfort from the endoscopy [24], those who were asymptomatic and at an early stage of EC/GC may have a lower compliance rate of endoscopy than those who were symptomatic. (3) In non-high-risk areas the endoscopists might be less experienced and trained, which may explain the lower early-detection rate and higher complication rate in non-high-risk areas compared with those in high-risk areas. Given the differences in EC/GC etiology between areas in China, the biology of EC/GC in high-risk population in low-risk region may also be different from those in average risk people in high-risk region. Novel pre-endoscopic screening tests should be developed in order to increase the detection rates and early-detection rates in non-high-risk areas [25]. Further evaluation on the efficacy of endoscopic screening in non-high-risk areas is still warranted [7].

Reducing mortality rate rather than screening itself is the optimal goal of screening programs for EC and GC. In our study, treatment of positive cases was not considered as intervention methods; however, we recommended each subject with advanced lesions for further treatment. And about 20% of those positive cases in our study refused further treatment because they identified themselves as “healthy” without any symptoms, and more than 95% of those untreated patients were in stage 0/I. The untreated proportion of positive cases in our study may diminish the beneficial effects of endoscopic screening. Chinese people traditionally conceal their sickness and are fearful of treatment [24]. Previous studies showed that there was a 30-fold higher risk of developing EC for patients with squamous severe dysplasia and over 80% high grade dysplasia of gastric mucosa could develop into GC [26, 27]. Endoscopic therapy or surgery has been shown to be effective and curative methods in early EC and GC [28, 29]. Standardized treatment of positive cases is necessary and further medical education should be carried out to improve treatment uptake in the screening population.

To the best of our knowledge, our study is the first multi-center cluster randomized trial of endoscopic screening for EC and GC in China in a well-organized population covering high-risk and non-high-risk areas in China. We recruited a total of 149,956 subjects in the trial and performed 37,922 standard endoscopies. The preliminary results may help to explore the benefit and harm of endoscopic screening. The large sample size could increase the power and the wide population from different areas of China may help generalize the trial findings. Our study may provide scientific evidence for development of screening procedures across different areas of the country. The follow-up of the trial is ongoing, which may allow us to study the influence of endoscopy on EC and GC mortality and incidence in different regions of China. There are also some limitations in our study. First, select bias could not be ignored due to the open-label recruitment and non-compliance rates in our trial. However, it is important to note that the baseline demographics did not differ between the intervention group and the control group. And the endoscopic compliance rate in our study was comparable or even higher compared with existed endoscopic screening of EC/GC [6, 8]. We may further evaluate potential confounders in our future analysis to adjust potential select bias. Second, to avoid unnecessary further biopsies and additional burden for participants in cancer screening, not all subjects were biopsied during the endoscopic screening. Even though, the adverse effects of endoscopic screening cannot be avoided in our study. Third, considering there are many differences in practice from western countries and underlying differences in the epidemiology of the disease, the screening strategies of upper gastrointestinal cancers in China may not be applicable in western countries.

## Conclusions

Our baseline results showed the ability to recruit and screen a large population at multiple centers in China. We found a higher detection rate and early-detection rate in high-risk areas than in non-high-risk areas of China. Further follow-up of this trial will provide valuable data regarding the effect of screening on EC and GC mortality.

## Supplementary information

**Additional file 1:** Supporting material.

## Data Availability

Currently, the data are not yet openly available. The study group welcomes potential collaboration to maximize use of existing resources. For more information, please contact the corresponding authors of this paper.

## Abbreviations

EC: Esophageal cancer
GC: Gastric cancer
NCC: National Cancer Center
SD: Standard deviation
OR: Odds ratio
CI: Confidence interval
ASR: Age-standardized rate

## References

[1] Bray F, Ferlay J, Soerjomataram I, Siegel RL, Torre LA, Jemal A (2018). Global cancer statistics 2018: GLOBOCAN estimates of incidence and mortality worldwide for 36 cancers in 185 countries. CA Cancer J Clin.

[2] Yoo KY (2008). Cancer control activities in the Republic of Korea. Jpn J Clin Oncol.

[3] Leung WK, Wu MS, Kakugawa Y, Kim JJ, Yeoh KG, Goh KL (2008). Screening for gastric cancer in Asia: current evidence and practice. Lancet Oncol.

[4] Wei WQ, Chen ZF, He YT, Feng H, Hou J, Lin DM (2015). Long-term follow-up of a community assignment, one-time endoscopic screening study of esophageal cancer in China. J Clin Oncol.

[5] Chen R, Liu Y, Song G, Li B, Zhao D, Hua Z (2020). Effectiveness of one-time endoscopic screening programme in prevention of upper gastrointestinal cancer in China: a multicentre population-based cohort study. Gut.

[6] Zhang X, Li M, Chen S, Hu J, Guo Q, Liu R (2018). Endoscopic screening in Asian countries is associated with reduced gastric cancer mortality: a meta-analysis and systematic review. Gastroenterology.

[7] Kim H, Hwang Y, Sung H, Jang J, Ahn C, Kim SG (2018). Effectiveness of gastric cancer screening on gastric cancer incidence and mortality in a community-based prospective cohort. Cancer Res Treat.

[8] Liu M, He Z, Guo C, Xu R, Li F, Ning T (2019). Effectiveness of intensive endoscopic screening for esophageal cancer in China: a community-based study. Am J Epidemiol.

[9] Li JY, Liu BQ, Li GY, Chen ZJ, Sun XI, Rong SD (1981). Atlas of cancer mortality in the People’s Republic of China. An aid for cancer control and research. Int J Epidemiol.

[10] Chen W, Zeng H, Chen R, Xia R, Yang Z, Xia C (2017). Evaluating efficacy of screening for upper gastrointestinal cancer in China: a study protocol for a randomized controlled trial. Chin J Cancer Res.

[11] Dawsey SM, Fleischer DE, Wang GQ, Zhou B, Kidwell JA, Lu N (1998). Mucosal iodine staining improves endoscopic visualization of squamous dysplasia and squamous cell carcinoma of the esophagus in Linxian. China Cancer.

[12] State Council of the People’s Republic of China. State Council issues plan to prevent chronic diseases. https://english.gov.cn/policies/latest_releases/2017/02/14/content_281475567482818.htm

[13] Kim BJ, Heo C, Kim BK, Kim JY, Kim JG (2013). Effectiveness of gastric cancer screening programs in South Korea: organized vs opportunistic models. World J Gastroenterol.

[14] Ogoshi K, Narisawa R, Kato T (2010). Endoscopic screening for gastric cancer in Niigata city. Jpn J Endosc Forum Dig Dis.

[15] Choi KS, Jun JK, Lee HY, Park S, Jung KW, Han MA (2011). Performance of gastric cancer screening by endoscopy testing through the National Cancer Screening Program of Korea. Cancer Sci.

[16] He Z, Liu Z, Liu M, Guo C, Xu R, Li F (2019). Efficacy of endoscopic screening for esophageal cancer in China (ESECC): design and preliminary results of a population-based randomised controlled trial. Gut.

[17] Zheng X, Mao X, Xu K, Lu L, Peng X, Wang M (2015). Massive endoscopic screening for esophageal and gastric cancers in a high-risk area of China. PLoS ONE.

[18] Hamashima C (2016). Benefits and harms of endoscopic screening for gastric cancer. World J Gastroenterol.

[19] Yoshimizu S, Hirasawa T, Horiuchi Y, Omae M, Ishiyama A, Yoshio T (2018). Differences in upper gastrointestinal neoplasm detection rates based on inspection time and esophagogastroduodenoscopy training. Endosc Int Open.

[20] Guan CT, Song GH, Li BY, Gong YW, Hao CQ, Xue LY (2018). Endoscopy screening effect on stage distributions of esophageal cancer: a cluster randomized cohort study in China. Cancer Sci.

[21] Riecken B, Pfeiffer R, Ma JL, Jin ML, Li JY, Liu WD (2002). No impact of repeated endoscopic screens on gastric cancer mortality in a prospectively followed Chinese population at high risk. Prev Med.

[22] Jun JK, Choi KS, Lee HY, Suh M, Park B, Song SH (2017). Effectiveness of the Korean National Cancer Screening Program in reducing gastric cancer mortality. Gastroenterology.

[23] Chen Q, Yu L, Hao CQ, Wang JW, Liu SZ, Zhang M (2016). Effectiveness of endoscopic gastric cancer screening in a rural area of Linzhou, China: results from a case-control study. Cancer Med.

[24] Zong L, Abe M, Seto Y, Ji J (2016). The challenge of screening for early gastric cancer in China. Lancet.

[25] Murphy G, McCormack V, Abedi-Ardekani B, Arnold M, Camargo MC, Dar NA (2017). International cancer seminars: a focus on esophageal squamous cell carcinoma. Ann Oncol.

[26] Wang GQ, Abnet CC, Shen Q, Lewin KJ, Sun XD, Roth MJ (2005). Histological precursors of oesophageal squamous cell carcinoma: results from a 13 year prospective follow up study in a high risk population. Gut.

[27] You WC, Li JY, Blot WJ, Chang YS, Jin ML, Gail MH (1999). Evolution of precancerous lesions in a rural Chinese population at high risk of gastric cancer. Int J Cancer.

[28] Pech O, May A, Manner H, Behrens A, Pohl J, Weferling M (2014). Long-term efficacy and safety of endoscopic resection for patients with mucosal adenocarcinoma of the esophagus. Gastroenterology.

[29] Minashi K, Nihei K, Mizusawa J, Takizawa K, Yano T, Ezoe Y (2019). Efficacy of endoscopic resection and selective chemoradiotherapy for stage i esophageal squamous cell carcinoma. Gastroenterology.

