# Supplementary material for: Initial results from a multi-center population-based cluster randomized trial of esophageal and gastric cancer screening in China
Source: BMC Gastroenterol. 2020 Nov 24;20:398. doi: 10.1186/s12876-020-01517-3 (PMC7686770; doi:10.1186/s12876-020-01517-3)
Supplement: Supplementary file 1 — Additional file 1: Supporting material. [file 12876_2020_1517_MOESM1_ESM.docx]

**Table S1. Profile of questionnaire used to define high-risk individuals in non-high-risk areas**

| **Items** | | **Contents** | **Point Criteria** | **Score** |
| --- | --- | --- | --- | --- |
| Smoking consumption | Questions were asked to determine whether the subjects ever smoked cigarettes. Current/ever smokers were further asked about their daily amount of cigarette and duration of smoking. | | Smoking at least 20 cigarettes per day and last for 10 years or more | 1 |
| Alcohol consumption | Questions were asked to investigate whether the subjects had lifetime history of alcohol consumption. Current /ever drinker were further asked about their detailed type of drinking, daily amount of alcohol and duration of drinking. | | Drinking at least 28g ethanol per day and last for 10 years or more | 1 |
| Dietary habits | Three items of dietary habits were investigated separately (salted food intake, fried food intake, and moldy food intake). For each item, subjects were asked about the frequency of intake. | | Eating salted food, fried food or moldy food at least once per week | 1 |
| Family history of cancer | Individuals were asked about family history of cancer, including specific cancer types and the corresponding relationship of the family member. | | Family history of upper gastrointestinal cancer in the immediate family and relatives within 3 generations. | 2 |
| Upper gastrointestinal symptoms | Subjects were asked questions regarding any of these clinical symptoms:1) pain in the chest/back when swallowing 2) vomiting 3) dysphagia 4) chronic heartburn or indigestion 5) unexplained weight loss 6) melena | | Having any of these upper gastrointestinal symptoms | 2 |
| Disease history | Questions were asked to determine whether the subjects had any history of upper gastrointestinal disease including gastritis, esophagitis or esophageal reflux | | Personal history of gastritis, esophagitis or esophageal reflux | 2 |
|  |  | | **High-risk individual^a^** | **>=2** |

**Table S2. Incidence rates of EC and GC in different areas of China in 2015 (per 100,000)**

| **Gender** | **Areas** | **EC** | |  | **GC** | |
| --- | --- | --- | --- | --- | --- | --- |
|  |  | **Crude rate** | **ASR^a^** |  | **Crude rate** | **ASR^a^** |
| **Male** | **High-risk** |  |  |  |  |  |
|  | Cixian | 94.4 | 113.6 |  | 77.6 | 91.9 |
|  | Linzhou | 80.8 | 64.4 |  | 95.2 | 76.4 |
|  | Wuwei | 65.1 | 53.3 |  | 172 | 138.7 |
|  | **Non-high-risk** |  |  |  |  |  |
|  | Changsha | 17.3 | 11.3 |  | 12.6 | 8.0 |
|  | Harbin | 11.4 | 6.2 |  | 28.4 | 15.4 |
|  | Luoshan | 21.9 | 23.2 |  | 68.6 | 77.2 |
|  | Sheyang | 51.0 | 28.8 |  | 66.0 | 37.6 |
| **Female** | **High-risk** |  |  |  |  |  |
|  | Cixian | 65.6 | 63.4 |  | 37.6 | 37.6 |
|  | Linzhou | 61.1 | 39.8 |  | 50.6 | 33.4 |
|  | Wuwei | 22.2 | 17.4 |  | 53.7 | 39.7 |
|  | **Non-high-risk** |  |  |  |  |  |
|  | Changsha | 2.4 | 1.4 |  | 8.4 | 5.2 |
|  | Harbin | 0.9 | 0.4 |  | 12.2 | 6.0 |
|  | Luoshan | 9.0 | 8.4 |  | 25.8 | 23.3 |
|  | Sheyang | 33.5 | 17.5 |  | 32.6 | 17.5 |

^a^ Age-standardized rate by World Segi's population

**Table S3. Detailed treatment options for positive cases screened from the cohort**

| **Therapeutic regimens** | **All**  ***N* (%)** | **High-risk areas**  ***N* (%)** | **Non-high-risk areas**  ***N* (%)** |
| --- | --- | --- | --- |
| Surgery | 382 (72.3) | 343 (71.8) | 39 (78) |
| Radiotherapy | 8 (1.5) | 8 (1.7) | 0 |
| Chemotherapy | 11(2.1) | 10 (2.1) | 1 (2) |
| Other treatment | 24 (4.5) | 22 (4.5) | 2 (4) |
| No treatment | 103 (19.5) | 95 (19.9) | 8 (16) |
